# Clinicopathological and radiographic features in 40 cats diagnosed with pulmonary and cutaneous *Rhodococcus equi* infection (2012–2018)

**DOI:** 10.1177/1098612X19886395

**Published:** 2019-11-14

**Authors:** Muhammad Waseem Aslam, Seng Fong Lau, Chelly Sze Lee Chin, Nur Indah Ahmad, Nor-Alimah Rahman, Krishnammah Kuppusamy, Sharina Omar, Rozanaliza Radzi

**Affiliations:** 1 Department of Veterinary Clinical Studies, Faculty of Veterinary Medicine, University Putra Malaysia, Serdang, Malaysia; 2 Department of Veterinary Pathology and Microbiology, Faculty of Veterinary Medicine, University Putra Malaysia, Serdang, Malaysia

**Keywords:** *Rhodococcus equi*, pulmonary, cutaneous, alveolar–interstitial

## Abstract

**Objectives:**

This retrospective study aimed to describe clinical manifestations, diagnostic options, radiological features, therapeutic plans and outcomes for cats infected with *Rhodococcus equi*.

**Methods:**

Forty cats aged between 2 months and 11 years old (median 6 months) that were definitively diagnosed with rhodococcosis between 2012 and 2018 were recruited in this study. Medical records were reviewed for information on signalment, history, clinical presentation, diagnostic testing, treatment plans and clinical outcomes.

**Results:**

Of the 40 cats, 36 showed the pulmonary form of the disease, with 35 (87.5%) presented with dyspnoea, while four cats presented with only cutaneous lesions. Mean body temperature was 38.7 ± 0.2^°^C. Dyspnoea was noted in 87.5% of the cats. Leukocytosis (58.3%) with band neutrophilia (83.3%), monocytosis (58.3%) and thrombocytopenia (55.5%) were prominent findings in the haematology reports. Hyperproteinaemia (61.1%) with hypoalbuminaemia (22.2%) and hyperglobulinaemia (63.8%) with a low albumin to globulin ratio (38.9%) were prominent features of blood biochemistry reports. An alveolar–interstitial pattern was noted in 75% of pre-thoracocentesis radiographs. Pleural effusion, hepatomegaly, thoracic lymphadenopathy and atelectasis of any lung lobe were seen in 88.9%, 75%, 41.7% and 36.1% of cats, respectively. Overall, the mortality rate was 67.5% in both forms.

**Conclusions and relevance:**

Clinicians should be aware that feline rhodococcosis manifests as a pulmonary disease at a much higher rate than previously reported. Further studies are required to address the epidemiology, pathophysiology, disease management and prognosis of feline rhodococcosis. The role of immunosuppression as a predisposing factor in feline rhodococcosis requires further investigation.

## Introduction

*Rhodococcus equi* is an aerobic, facultatively intracellular Gram-positive, coccobacillus.^1^ It is a catalase-positive, oxidase-negative and mostly urease-positive bacterium,^2,3^ which can grow easily on ordinary media such as blood agar^4^ at 30–37ºC^3^ at a pH range of 7.0–8.5 (optimum 7.5).^5^ It can form coalescent (24 h incubation) to smooth glistening or mucoid colonies (after 48 h incubation) from 2 to 4 mm in diameter, which can be salmon pink to red-coloured, depending on the length of incubation period, although this is not an absolute identification finding.^2,5,6^ Nutritional requirements are very simple and herbivore manure can provide abundant organic acids such as propionate and acetate.^7^ The organism was formerly known as *Corynebacterium equi* and *Mycobacterium equi*.^8,9^ Currently, this bacterium is classified in the family Nocardiaceae of the order Actinomycetales, but a proposal was made in 2013 to reclassify it as *Prescottia equi*.^10^

*R equi* is distributed worldwide with a highly variable pattern.^2^ This bacterium has zoonotic potential and has been increasingly reported in immunocompromised human patients.^11–13^ The pathogenicity of *R equi* infection in horses has been extensively studied and well documented. Dogs, cats and other domestic and wild animals are also susceptible to this ubiquitous bacteria.^14^ Virulent *R equi* survives and multiplies in alveolar macrophages and polymorphonuclear cells by inhibiting maturation and acidification of the phagosome.^4^

The major routes of transmission are inhalation and ingestion.^2^ The lungs have been described as a predilection site in foals manifesting chronic pyogranulomatous bronchopneumonia.^15^ The most common abdominal manifestation is diarrhoea associated with ulcerative enterotyphlocolitis.^16,17^ Extrapulmonary disorders (EPDs), such as ulcerative lymphangitis, pyogranulomatous lymphadenopathies, pyogranulomatous hepatitis, peritonitis, pericarditis, granulomatous meningitis, subcutaneous abscess, immune-mediated polysynovitis, haemolytic anaemia, osteomyelitis and septic arthritis, have also been reported in foals.^18^ Manifestation of EPDs with primary predilection sites of infection has been correlated with a poor survival rate of 43%, as compared with 82% of cases without EPDs.^16^

In humans, pneumonia is the most common manifestation along with EPDs, as outlined by Khurana.^2^ For example, abscess of the brain,^19^ meninges, peritoneum and thyroid gland; fever; diarrhoea; lymphadenitis; pericarditis; polysynovitis; osteoarthritis; osteomyelitis; colonic polyps;^20^ mass in the lungs; granulomatous mastitis;^21^ and endophthalmitis specifically in immunocompromised patients have been documented. In another study comparing the mortality rate of rhodococcosis in human patients, the highest mortality rate was reported to be in people with HIV, followed by immunocompromised non-HIV-positive patients with rhodococcosis, and the lowest mortality rate was in immunocompetent patients.^22^ The overall mortality rate in human patients has been reported as 25%.^23^

There is a paucity of information and documented data on *R equi* infection in cats. Three studies on feline pulmonary rhodococcosis have been reported from Italy, the USA and Australia.^4,24,25^ These studies characterised *R equi* infection in cats and compared it with the disease reported in a highly susceptible host; that is foals. Two previous studies reported feline pulmonary rhodococcosis based on the history, clinical presentation and cytological evaluation of the tracheal exudate without any necropsy confirmation. Immunosuppression was suspected in these cats based on the intestinal lymphoma in one cat and clinical presentation only in another cat.^4,24^ Radiographic features of feline pulmonary rhodococcosis have not been described to date because of the limited number of cases reported. In foals, radiographic findings include an unstructured interstitial pattern, various combinations of the alveolar pattern, nodular or cavitary lesions and lymphadenopathy, especially tracheobronchial and pleural effusion. Furthermore, the severity of radiographic finding significantly correlated with the survival of the foals infected with *R equi*.^26^

The aim of this study is to describe the clinical manifestations, diagnostic options, radiological features, therapeutic plans and outcome for cats infected with *R equi*.

## Materials and methods

Feline patients diagnosed with *R equi* infection from 2012 to 2018 were recruited. Of these cats, all met the inclusion criteria of the study: (1) diagnosed with pulmonary or cutaneous rhodococcosis; and (2) returned at least once or were boarded for follow-up during the course of treatment.

Data available at the hospital including patient signalment, history, clinical presentation, haematology, serum biochemistry, radiographs, cytology and additional information, such as serology results of feline infectious peritonitis (FIP) antibody, feline immunodeficiency virus antibody and feline leukaemia virus antigen, were obtained. Radiographs were reviewed by two veterinarians to reach a consensus. Severity criteria for radiographic pulmonary patterns were adopted from a study by Crisi et al,^27^ where bronchial signs were categorised as mild, moderate and severe based on the first-, second- and third-generation bronchi visibility. Alveolar patterns were categorised as mild when there was presentation of isolated fluffy infiltrates, moderate when the pattern was well defined with air bronchograms and severe with lobar signs. Interstitial changes were categorised as mild when an interstitial framework was visible but when a bronchial pattern was suspected as well the moderate category included an interstitial framework distinguishable from bronchial; and the severe category was recorded for undisputed reticular interstitial patterns. Visualisation of a vascular pattern was aided by the relative enlargement of pulmonary vessels between the artery and vein.

Bacteria were cultured on blood agar with 5% defibrinated horse blood with an incubation period of 24–48 h at 37ºC, from pleural effusion drained by thoracocentesis and/or a chest tube, and sterile cotton swab samples collected from lungs by thoracotomy for 36 cats with the manifested pulmonary form of the disease. In the remaining four cats manifesting the cutaneous form of the disease, samples were collected through fine-needle aspiration (FNA) of abscess and sterile cotton swabs from contaminated open wounds. Large, mucoid, non-haemolytic salmon-pink colonies, suggestive of *R equi*, were processed further by performing conventional biochemical tests for identification of bacteria.^28^

Haematology and selected blood biochemistry parameters were evaluated. Antibiotic susceptibility was tested in some cases by the Kirby Bauer technique.^29^ Thirty-seven cats in this study received antibiotic(s), and were assessed for recovery and outcome based on the records of subsequent visits to the University Veterinary Hospital (UVH). Seventeen combinations of antibiotics are outlined in the ‘Results’ section for the pre- and post-diagnosis phases of disease management, and the outcome is shown with prominent circumstances that have been linked to these cases. Owners were also contacted by the hospital via telephone to obtain information on the post-treatment outcomes in cases where they did not return for follow-up appointments. All data are presented as descriptive statistics.

## Results

### Signalment and history

**Figure 1:**
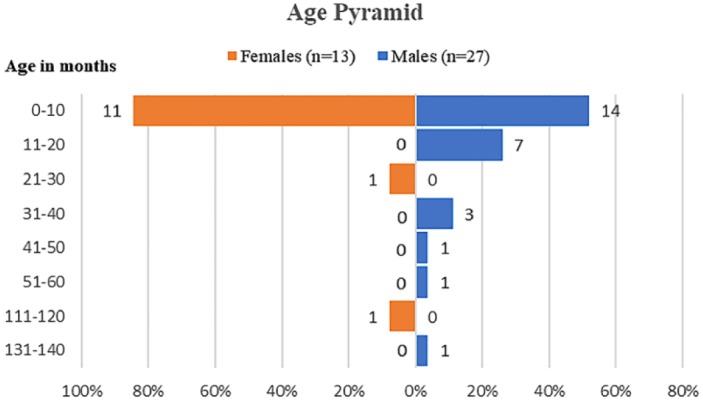
Age distribution of the cats

Forty cats confirmed positive (2012–2018) for *R equi* culture (27 males, 13 females), aged from 2 months to 11 years (median 6 months), were recruited in this study (Figure 1). Of these 40 cats, 30 were domestic shorthairs, four were Persian crosses, two were domestic longhairs, two were pure Persians, one was a Turkish Angora and one was a Maine Coon. Thirty-six cats were presented with the pulmonary form of *R equi* infection and showed acute respiratory distress (starting <3 weeks before presentation), with a dubious history of trauma. The other four cats presented with the cutaneous form, reported to manifest lesions, either non-resolving by empirical treatment or chronic in nature (>3 weeks), without any history of trauma or injuries. Of these 36 cats with pulmonary rhodococcosis, two cats underwent therapeutic thoracotomy, one died 7 days postoperatively and the other recovered. Another three cats with acute respiratory presentation died without any response to resuscitation and consent was obtained from the owners to perform a necropsy.

### Clinical findings

The mean body temperature during patient presentation was 38.7 ± 0.2^º^C (n = 35). At clinical examination, respiratory signs were the predominant findings, as compared with other non-specific clinical signs in sick cats. Thirty-five of the 36 cats presented with respiratory distress and five cats from this group had concurrent coughing. Only one cat did not show respiratory distress, but was reported to have a chronic cough. The most frequent signs observed were dyspnoea (87.5%), abdominal breathing (65%), anorexia (65%), tachypnoea (47.5%), dehydration (35%) and open-mouth breathing (32.5%) (Table 1). Lung auscultation revealed crackles as the most predominant finding (27.5%). Other non-specific signs documented were weight loss, coughing, lethargy, pyrexia, ocular/nasal discharge, vomiting and diarrhoea. Cyanosis of the mucous membrane was also noted in one case. Cutaneous lesions reported in four cats and topographical locations were soft tissue swellings around the neck area; left distal carpal, proximal mediocaudal aspect of the left forelimb; right distal radius and ulna up to digits; and open wounds around the caudoventral chest area with an intact thoracic wall.

**Table 1:** Clinical signs of 40 cats infected with *Rhodococcus equi*

Clinical signs	n (%)
Dyspnoea/respiratory distress	35 (87.5)
Abdominal breathing	26 (65.0)
Anorexia/hyporexia	26 (65.0)
Tachypnoea	19 (47.5)
Dehydration	14 (35.0)
Open-mouth breathing	13 (32.5)
Crackles on thoracic auscultation	11 (27.5)
Lethargy	8 (20.0)
Weight loss	7 (17.5)
Coughing	5 (12.5)
Pyrexia	5 (12.5)
Cutaneous lesions	4 (10)
Ocular/nasal discharge	2 (5.0)
Diarrhoea	2 (5.0)
Cyanotic mucous membranes	1 (2.5)
Vomiting	1 (2.5)

### Haematological and biochemical results

Mean and median haematological and biochemical results are presented in Table 2. Of the 36 available haematological results, the most significant abnormalities were left-shift neutrophilia (83.3%), neutrophilia (66.7%), leukocytosis (58.3%) and monocytosis (58.3%). Thrombocytopenia was noted in 55.5% of samples and confirmed with blood smears. Changes in protein levels were the most significant findings, which also altered the albumin to globulin (A:G) ratio (median 0.6). The median albumin level was calculated as 26.9 g/l (range 25–40 g/l), while the median globulin level was 48.8 g/l (range 25–45 g/l) and was elevated in 63.8% of the samples.

**Table 2:** Physiological, biochemical and haematological parameters of 36 cats infected with *Rhodococcus equi*

Parameters (number of samples)	Normal reference range	Mean ± SE	Median	n (%) with value > upper RL	n (%) with value < upper RL
Body temperature (^°^C) (n = 35)	38–39	38.7 ± 0.15	38.8	12 (34.3)	7 (20)
PCV (l/l) (n = 36)	0.24–0.45	0.295 ± 0.009	0.3	0 (0)	4 (11.1)
CWCC (×10^9^/l) (n = 36)	5.50–19.5	31.25 ± 4.268	22.15	21 (58.3)	0 (0)
Segmented neutrophils (×10^9^/l) (n = 36)	2.50–12.5	23.57 ± 3.290	14.97	24 (66.7)	0 (0)
Band neutrophils (×10^9^/l) (n = 36)	<0.30	0.992 ± 0.184	0.585	30 (83.3)	0 (0)
Lymphocytes (×10^9^/l) (n = 36)	1.5–7.0	4.150 ± 0.734	3.02	4 (11.1)	7 (19.4)
Monocytes (×10^9^/l) (n = 36)	0.20–0.80	1.434 ± 0.230	0.94	21 (58.3)	0 (0)
Eosinophils (×10^9^/l) (n = 31)	0.1–1.5	1.294 ± 0.229	0.98	11 (35.4)	0 (0)
Platelets (×10^9^/l) (n = 36)	300–700	321.7 ± 39.65	266	4 (11.1)	20 (55.5)
Na^+^ (mmol/l) (n = 30)	146–156	149.5 ± 0.833	150.4	2 (6.7)	4 (13.3)
K^+^ (mmol/l) (n = 30)	3.90–5.50	05.00 ± 0.159	4.85	6 (20)	2 (6.7)
Cl^–^ (mmol/l) (n = 30)	110–132	111.7 ± 1.041	113.05	0 (0)	6 (20)
Phosphorus (mmol/l) (n = 3)	1.10–2.80	03.44 ± 0.311	3.28	3 (100)	0 (0)
Urea (mmol/l) (n = 35)	3.1–10.0	8.766 ± 0.870	7.8	8 (22.9)	0 (0)
Creatinine (µmol/l) (n = 35)	60–193	90.31 ± 7.755	82	0 (0)	6 (17.1)
TBIL (µmol/l) (n = 2)	1.7–17.0	1.305 ± 0.405	1.305	0 (0)	1 (50)
ALT (U/l) (n = 35)	10–90	49.43 ± 5.393	41	2 (5.7)	0 (0)
ALP (U/l) (n = 9)	<80	43.44 ± 18.57	23	2 (22.2)	0 (0)
GGT (U/l) (n = 2)	<6.0	13.50 ± 08.50	13.5	1 (50)	0 (0)
CK (U/l) (n = 2)	<300	376.0 ± 25.00	376	2 (100)	0 (0)
AST (U/l) (n = 2)	<60.0	261.5 ± 89.50	261.5	2 (100)	0 (0)
TP (g/l) (n = 36)	55–75	78.49 ± 1.768	79.15	22 (61.1)	0 (0)
Albumin (g/l) (n = 36)	25–40	28.34 ± 0.849	26.9	1 (2.8)	8 (22.2)
Globulin (g/l) (n = 36)	25–45	50.14 ± 1.463	48.8	23 (63.8)	0 (0)
A:G (n = 36)	0.5–1.4	0.577 ± 0.022	0.6	0 (0)	14 (38.9)

RL = reference limit; PCV = packed cell volume; CWCC = complete white cell count; TBIL = total bilirubin; ALT = alanine transaminase; ALP = alkaline phosphatase; GGT = gamma glutamyl transferase; CK = creatine kinase; AST = aspartate transaminase; TP = total protein; A:G = albumin:globulin ratio

### Radiographic findings

**Figure 2:**
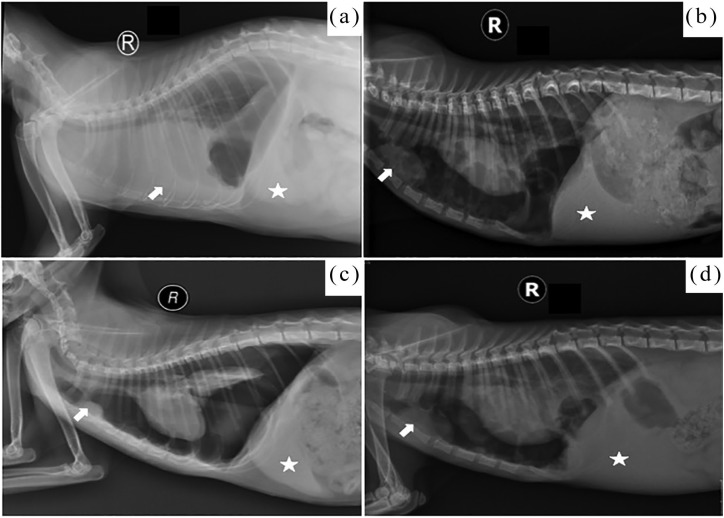
Thoracic radiographs of four cats showing different types of pathologies. Enlargement of the retrosternal lymph node (white arrow) was seen in (b–d) and hepatomegaly (white star) was noted in all four cats. (a) Pleural effusion is indicated by an arrow and alveolar–interstitial pattern of the lungs with air pocket/pneumatocoele below the level of accessory lung lobe. (b) Pneumothorax, cavitary lesion in mid thorax cranial to the diaphragm. (c) Pneumothorax with atelectasis of the the right caudal and accessory lung lobes. (d) Pneumothorax with pneumatocoele just caudal to the apex of the heart and cranial to the diaphragm

The frequency of various pathologies and outcomes of feline rhodococcosis in these 40 cats are summarised in Table 3. The most prominent abnormality noted in thoracic radiographs was pleural effusion (88.9%). The second most common abnormality was hepatomegaly (75%). Atelectasis and/or consolidation of any lung lobe, thoracic lymphadenopathy (retrosternal lymph node and/or tracheobronchial lymph node), cavitary lesions and fluid/abnormal soft tissue opacity of lungs and pneumotho-rax were notable findings on radiographs (Figure 2). Radiographs of cutaneous lesions showed massive soft tissue swelling with air pockets between tissue/fascia and irregular margins without any bony involvement.

**Table 3:** Summary of pathologies and outcomes in a maximum of 40 cases of cats infected with *Rhodococcus equi*

Parameter (total number of cats)	n (%)
Pleural effusion (n = 36)	32 (88.9)
Hepatomegaly (n = 40)	30 (75)
Thoracic lymphadenopathy (n = 36)	15 (41.7)
Atelectasis of any lung lobe (n = 36)	13 (36.1)
Consolidation of any lung lobe (n = 36)	11 (30.6)
Cavitary or mass opacity lesion(s) (n = 36)	10 (27.8)
Pneumothorax (n = 36)	6 (16.7)
Died (n = 40)	23 (57.5)
Recovered (n = 40)	13 (32.5)
Euthanased (n = 40)	4 (10)
Total dead cases (n = 40)	27 (67.5)

Abnormal radiographic patterns for all cats are summarised in Table 4. In pre-thoracocentesis radiographs, severe alveolar, mild interstitial and mixed (alveolar–interstitial) patterns were the predominant findings and noted in 55.5%, 50% and 86.1% of cases, respectively. The most prominent post-thoracocentesis patterns were severe alveolar (58.3%), moderate interstitial (41.7%) and alveolar–interstitial (52.8%). Mixed radiographic patterns (97.1%) were the predominant findings in post-thoracocentesis radiographs. A solitary pattern was not significant in both pre- and post-thoracocentesis radiographs.

**Table 4:** Summary of the pre- and post-thoracocentesis radiographic patterns of 36 cats infected with the pulmonary form of *Rhodococcus equi*

	Pre-thoracocentesis findings	Post-thoracocentesis findings
Bronchial		
Mild	3 (8.3)	11 (30.6)
Moderate	1 (2.8)	3 (8.3)
Severe	0 (0)	0 (0)
Alveolar		
Mild	0 (0)	4 (11.1)
Moderate	16 (44.4)	10 (27.8)
Severe	20 (55.5)	21 (58.3)
Interstitial		
Mild	18 (50)	14 (38.9)
Moderate	5 (13.9)	15 (41.7)
Severe	5 (13.9)	4 (11.1)
Mixed pattern	31 (86.1)	33 (91.7)
Bronchioalveolar	2 (5.6)	1 (2.8)
Alveolo–interstitial	27 (75)	19 (52.8)
Bronchointerstitial	0 (0)	0 (0)
Bronchial + Alveolar + Interstitial	2 (5.6)	13 (36.1)
Solitary pattern		
Bronchial	0 (0)	0 (0)
Alveolar	5 (13.9)	1 (2.8)
Interstitial	0 (0)	0 (0)

Values are n (%)

### Sampling and sample properties

Diagnostic and/or therapeutic thoracocentesis was performed in 31/36 cats presented with the pulmonary form of the disease. Along with thoracocentesis in the initial management, sterile cotton swab samples were also collected from the lungs of two cats that underwent therapeutic thoracotomy. Post-mortem lung, liver and kidney tissue samples were collected from three cats. An FNA sample was collected from a large abscess on the neck region of one cat, and sterile cotton swab samples of contaminated wounds and an impression smear were collected from the other three cats with the cutaneous form of the disease. From the available data of 40 cats, 27 records had detailed information regarding the physicochemical properties of the samples submitted for cytology and bacterial culture.

**Figure 3:**
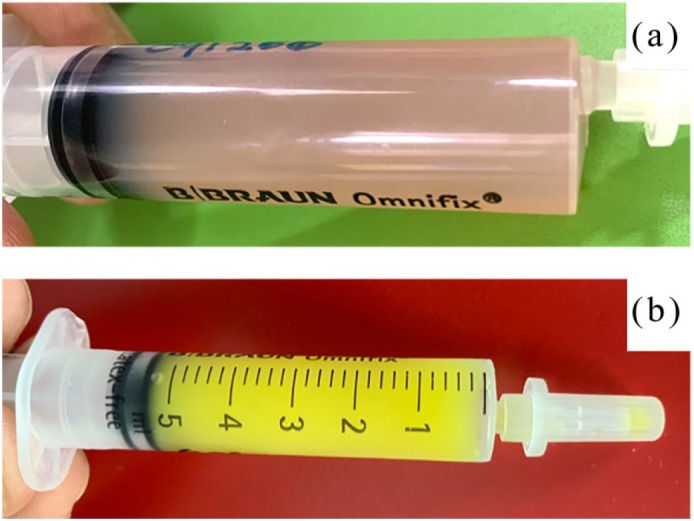
(a) Light-pinkish appearance of the pleural effusion sample from a cat positive for *Rhodococcus equi*. (b) Gel-like pale yellow appearance of another pleural effusion sample from a cat positive for *R equi*

Of these 27 samples, 26 showed pleural effusion and one sample was of a pus-like discharge, collected by thoracocentesis and by FNA of a cutaneous lesion, respectively. Regarding the appearance of these samples, six were pale yellow, five light yellow, four a creamy light yellow, four a milky light yellow, four a light pink colour (Figure 3a), one was a pale yellow and gel-like (Figure 3b), one was light brown, one was light green and one was pale grey. Turbidity was graded on a scale of 0 to 4+, with 0 being crystal clear and 4+ being so turbid that newsprint could not be read through the tube.^30^ Twenty samples were graded as 3+ and seven as 4+. The pH of these samples ranged from 6 to 8, the protein level ranged from 3+ (300 mg/dl) to 4+ (>1000 mg/dl) and the specific gravity ranged from 1.021 to 1.041.

### Cytological analysis

**Figure 4:**
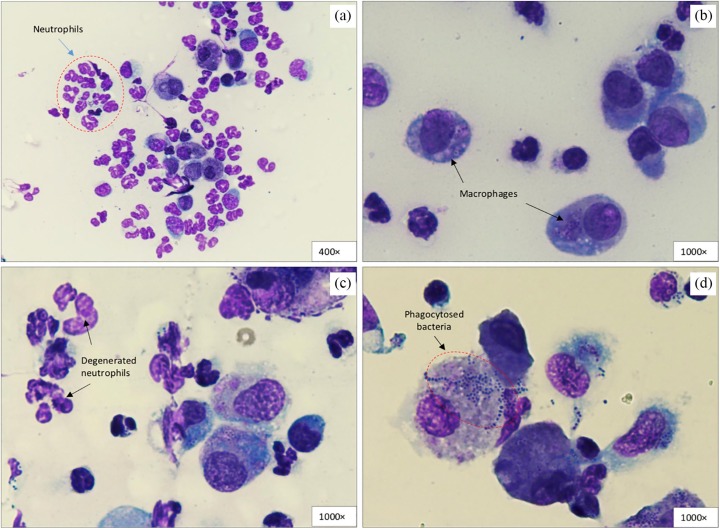
Cytological smear from an exudate of a massive soft tissue swelling of the left forelimb. Neutrophilic pyogranulomatous inflammation was diagnosed. (a) Neutrophils with poor cytoplasmic detail encircled with a red dotted line; a second population of large-sized cells consists of macrophages with phagocytosed coccobacillus bacteria (× 400). (b) From the same cat as in (a), large macrophages containing numerous purple cocci to coccobacilli (arrows) in the cytoplasm (× 1000). (c) Similar bacterial population within neutrophils and macrophages. Degenerated neutrophils had been labelled (× 1000). (d) Phagocytosed bacteria within the foamy cytoplasm of a giant macrophage encircled with a red dotted line (× 1000)

Cytology was performed for 28 samples submitted as pleural fluid and sterile cotton swabs from the lungs and infected wounds. Pyogranulomatous inflammation was the conclusive finding in all of the samples and phagocytosed bacteria (coccobacillus/cocci/mixed type) was detected in 86% of samples (n = 24). Neutrophils and macrophages were the most abundant cell types identified in cytological studies (Figure 4).

### Therapy and outcomes

Antibiotic susceptibility tests were performed for commonly used antibiotics in 19 of the cases (Table 5). Resistance to cephalexin was reported as the highest (14 samples were resistant). Clindamycin (n = 2) and tetracycline (n = 4) demonstrated 100% resistance. Metronidazole and potentiated amoxicillin also showed poor susceptibility against the relevant strain of *R equi*. Azithromycin, erythromycin and gentamicin showed 100% susceptibility, but the sample size was limited. Enrofloxacin (81.3%) and marbofloxacin (75%) possessed sufficient susceptibility and was tested in 16 samples.

**Table 5:** Summary of antibiotic susceptibility tests for a maximum of 19 cases of cats infected with *Rhodococcus equi*

Antibiotic (number of samples)	Disk content (µg)	Resistance status (R+I)	Susceptibility status (S)
Cephalexin (n = 14)	30	100	0
Clindamycin (n = 2)	2	100	0
Tetracycline (n = 4)	30	100	0
Metronidazole (n = 8)	5	87.5	12.25
Amoxicillin–clavulanic acid (n = 19)	30	68.4	31.6
Amoxicillin (n = 6)	10	66.6	33.3
Sulfamethoxazole/trimethoprim (n = 5)	25	60	40
Marbofloxacin (n = 16)	5	25	75
Enrofloxacin (n = 16)	5	18.75	81.25
Azithromycin (n = 4)	15	0	100
Ceftriaxone (n = 1)	30	0	100
Erythromycin (n = 3)	15	0	100
Gentamicin (n = 2)	10	0	100

Based on the available data about the use of antibiotic(s) with/without combination (17 combinations altogether), 37 cats received antibiotics and three cats died on acute presentation. From Table 6, it is not possible to conclude on any successful combination in pre- or post-diagnostic management of feline rhodococcosis for a satisfactory outcome. It was mainly dependent on the susceptibility of bacterial strain (virulent or avirulent) to any antibiotic(s) from pre- and post-diagnosis combinations that have been used in reported cases. Although 25% of samples were resistant against marbofloxacin and the sample size was quite small for azithromycin, these antibiotics appeared to be reasonably effective against reported cases, along with rifampin. Unfortunately, none of the samples were tested against rifampin.

**Table 6:** Summary of antibiotic(s) usage and the dose range in 37 cats infected with *Rhodococcus equi*

	Pre-diagnosis (antibiotic[s])	Post-diagnosis (antibiotic[s])	No. of cats (n = 37)	Outcome (n = 37)	Remarks
1	Amoxicillin–clavulanic acid	–	3	3 died	One owner refused boarding; 2 died in pre-diagnostic management
2	Amoxicillin–clavulanic acid	Azithromycin + rifampin	3	2 recovered, 1 died	Of 2 recovered cats, 1 went through a course of enrofloxacin + azithromycin before presentation. Other bacteria were susceptible to azithromycin, but infection relapsed after apparent recovery of this cat, possibly because of poor compliance, and the cat died 9 weeks after initial diagnosis because of a similar presentation
3	Marbofloxacin	–	2	1 recovered, 1 died	One cat died in pre-diagonstic management, while the other went through thoracotomy, and bacteria susceptible to marbofloxacin
4	Marbofloxacin	Azithromycin	1	1 died	Went through thoracotomy but died within 7 days of postoperative management of infection
5	–	Gentamicin + rifampin	1	1 euthanased	Owner refused post-diagnosis management
6	Marbofloxacin + metronidazole	–	3	3 died	Two cats died during pre-diagnostic management; third cat died during long-term (4 weeks) management post-diagnosis
7	Marbofloxacin + metronidazole	Switched to azithromycin + rifampin	1	1 recovered	Bacteria susceptible to azithromycin
8	Amoxicillin–clavulanic acid + metronidazole	Switched to azithromycin + rifampin	8	5 recovered, 2 died, 1 euthanased	Of the two cats that died, 1 was FeLV positive. Infection relapsed after an apparently full recovery in 1/5 recovered cats, possibly because of poor compliance, and the cat died 10 weeks after the initial diagnosis because of a similar presentation
9	Amoxicillin–clavulanic acid + metronidazole	Switched to marbofloxacin + rifampin	2	2 recovered	Bacteria was susceptible to marbofloxacin in both cases
10	Amoxicillin–clavulanic acid + metronidazole	–	4	4 died	All cats died during pre-diagnostic management of infection
11	Amoxicillin–clavulanic acid + azithromycin	Rifampin added and amoxicillin–clavulanic acid stopped	1	1 euthanased	Poor response to treatment during long-term management (4 weeks)
12	Amoxicillin–clavulanic acid + metronidazole + azithromycin	Rifampin added and amoxicillin–clavulanic acid and metronidazole stopped	2	1 recovered, 1 died	Dead cat had a high FCoV antibody titre and a low A:G (0.5), supporting clinical signs
13	Amoxicillin–clavulanic acid + marbofloxacin + azithromycin	Rifampin added and amoxicillin–clavulanic acid stopped	1	1 recovered	–
14	Amoxicillin–clavulanic acid + metronidazole + marbofloxacin	Rifampin added and amoxicillin–clavulanic acid and metronidazole stopped	2	1 recovered, 1 died	Poor response to antibiotics seen in the dead cat and bacteria were resistant to marbofloxacin
15	Amoxicillin–clavulanic acid + metronidazole + marbofloxacin	Switched to azithromycin and rifampin	1	1 euthanased	Poor response to antibiotics and chest tube management, although bacteria were susceptible to azithromycin
16	Amoxicillin–clavulanic acid + metronidazole + marbofloxacin	–	1	1 recovered	Eight weeks of therapy and bacteria were susceptible to marbofloxacin
17	Amoxicillin–clavulanic acid + metronidazole + enrofloxacin	–	1	1 died	Died during pre-diagnostic management

Amoxicillin–clavulanic acid: 12.5–20 mg/kg q12h; metronidazole: 10–15 mg/kg (lower dosage q8h and higher dosage q12h); marbofloxacin: 2–4 mg/kg q24h (higher dosage for susceptible bacteria); enrofloxacin: 5 mg/kg q24h for a maximum of 3 days; azithromycin: 10 mg/kg q24h used for a maximum of 8 weeks; rifampin: 10 mg/kg q24h used for a maximum of 8 weeks (with on and off adverse effects in three cases, such as anorexia and vomiting possibly related to hepatotoxicity)

FeLV = feline leukaemia virus; FCoV = feline coronavirus; A:G = albumin to globulin ratio

Nineteen cats were started on double therapy before a definitive diagnosis was reached, as shown in Table 6 (combinations 6–11). The most successful combinations were combinations 8 and 9 (Table 6), which were used in 10 cats where initial management was started with amoxicillin–clavulanic acid and metronidazole, and later switched to either azithromycin + rifampin or marbofloxacin + rifampin; seven cats recovered on this combination, although the bacteria were susceptible to azithromycin and marbofloxacin in this group. Combination 10 (Table 6) provided similar premedication, but all cats died before definitive diagnosis. In combination with number 6, all cats died as a result of the reasons described in the ‘Remarks’ column of Table 6, but the same pre-diagnostic combination when switched with azithromycin and rifampin, led to the recovery of one cat.

Eight cats were started on triple therapy during pre-diagnosis management, and in this group four cats recovered, one was euthanased, one was resistant to marbofloxacin, one was carrying concurrent FIP based on weak evidence (elevated antibody titre, low A:G ratio, supportive clinical signs) and one died during initial disease management. Furthermore, 10 cats were started on single therapy during the pre-diagnosis management. Only three cats recovered; of these three, one underwent thoracotomy and bacteria were also susceptible to marbofloxacin, two were switched to azithromycin and rifampin where one sample was susceptible to azithromycin, and the other cat was managed with enrofloxacin and azithromycin by another vet before presentation to UVH.

**Figure 5:**
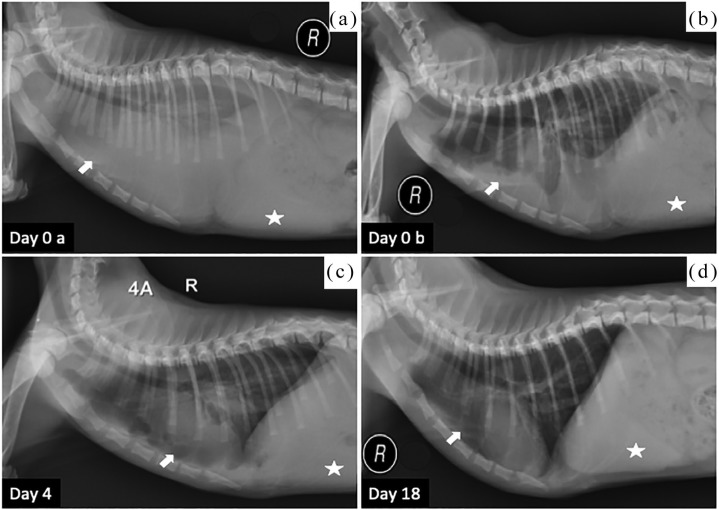
Therapeutic changes seen on thoracic radiographs of a cat diagnosed with pulmonary rhodococcosis. Hepatomegaly was (white star) was noted in all four radiographs. (a) Day 0_a_: massive pleural effusion (white arrow) with air bronchogram. (b) Day 0_b_: post-thoracocentesis radiograph with a reduced amount of fluid and lobar sign. (c) Day 4 radiograph showed improvement with a small amount of fluid (arrow). (d) Day 18: complete clearance of fluid from chest cavity

**Figure 6:**
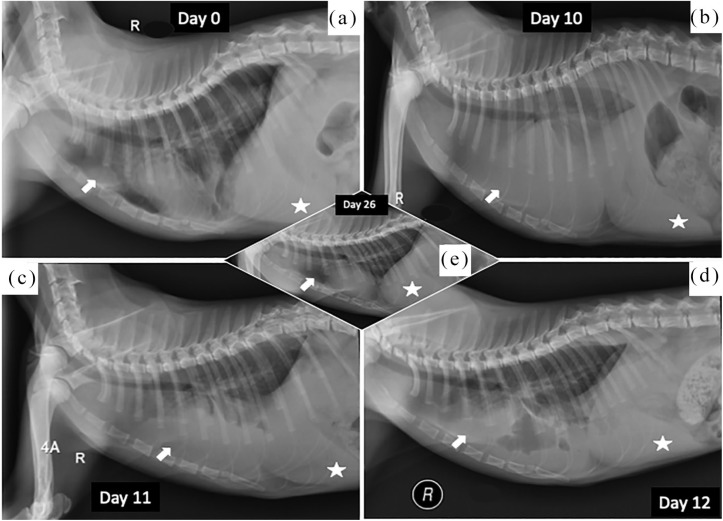
Therapeutic changes seen on the thoracic radiographs of a cat diagnosed with pulmonary rhodococcosis. Hepatomegaly was (white star) was noted in all radiographs. (a) Day 0 radiograph showed retracted lung lobes with an enlarged retrosternal lymph node (white arrow). The cat was treated as an outdoor patient and was asked to attend follow-up. (b) Arrow showing pleural effusion and prominent lobar sign noted at day 10. Thoracocentesis was performed at day 10. (c,d) Post-thoracocentesis radiographs showed a reduced amount of fluid (white arrow) in the chest cavity. (e) Day 26 radiograph showed a complete clearance of fluid from the chest cavity

The outcomes of the patients recruited to this study are presented in Table 3. Of 40 cats, 23 died during the initial or long-term management of the pulmonary and cutaneous forms of the infection. Four cats were euthanased in both categories. Only 1/4 cats with the cutaneous form recovered. Another cat from this group died during the course of treatment and was tentatively diagnosed with FIP, with hepatomegaly seen on the radiographs. One cat with the cutaneous form was euthanased and one did not respond to therapy. The overall mortality rate was 67.5% for both forms of the disease. The average duration of antibiotic therapy was 6 weeks and improvement was monitored with radiographs (Figures 5 and 6).

### Post mortem and histopathology results

Pyogranulomatous lesions on lung lobes, pyothorax, nutmeg liver and congested kidneys were reported in all three cats that underwent post-mortem examination. Atelectasis of any lung lobe was seen in 2/3 cats, while the third cat had a pyogranulomatous mass (5 cm × 3 cm) attached to the heart, lungs and rib cage. Hepatomegaly and gall bladder distension were also documented in one cat.

Infiltration of inflammatory cells (neutrophils, macrophages and plasma cells) was the most prominent finding in histopathology samples of lung tissue from all three cats. Hepatocellular necrosis with inflammatory cells and generalised renal tubular necrosis was reported in 2/3 samples. Thickening of interalveolar septa was noted in one of the three samples. Generalised congestion of lung tissue and intrahistiocytic coccobacillus bacteria was noted in 2/3 specimens.

## Discussion

In this study, the results revealed that the pulmonary manifestation was the most common finding and was seen in 90% of cats infected with *R equi*. This manifestation contradicts previous studies, where it was believed that the most common route of transmission was transcutaneous infection from wounds manifesting as the cutaneous form of the disease, followed by the aerogenous route, which can disseminate to the body cavities and organs via the haematogenous route.^4,12,24^ Sex and age distribution (Figure 1) and radiographic findings have never been correlated with the natural host in previous feline studies because of the limited number of cases.

**Figure 7:**
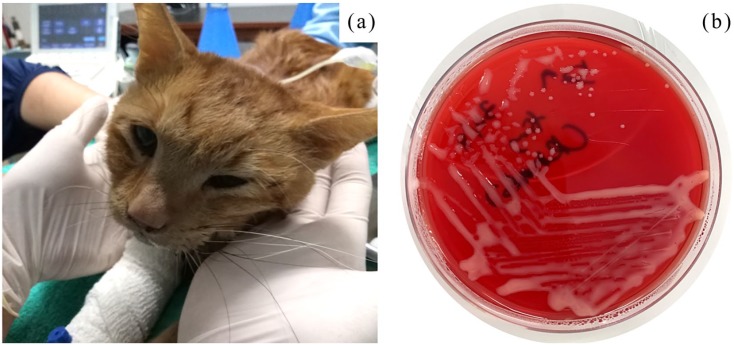
(a) Presentation of a cat diagnosed with *Rhodococcus equi* during ultrasound-guided thoracocentesis and pleural effusion sample collection. (b) Pure growth of *R equi* on blood agar

Kittens up to the age of 10 months with typical dyspnoic presentation and concurrent septic pleural effusion (Figure 7a), with band neutrophilic/neutrophilic and monocytic leukocytosis, thrombocytopenia and low A:G ratio, can be potential candidates for carrying the pulmonary form of *R equi* infection. Isolation and identification of bacteria can be performed by culturing samples on 5% defibrinated horse blood agar by incubating for 24–48 h at 37ºC for a definitive diagnosis. Mucoid, non-haemolytic salmon-pink colonies (Figure 7b) are suggestive of *R equi*, which can be confirmed with commercial or conventional biochemical tests.

**Figure 8:**
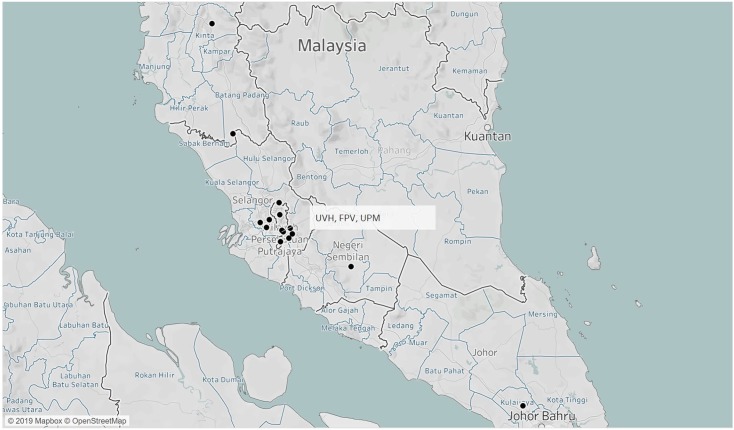
Origin locations of the cats diagnosed with *Rhodococcus equi* at University Veterinary Hospital (UVH), Fakulti Perubatan Veterinar (FPV) and Universiti Putra Malaysia (UPM)

The tropical environment of Malaysia with a relatively uniform temperature throughout the year, ranging from 24ºC to 28ºC supports the growth of this organism.^31^ Although this bacterium grows well in low humidity, the humidity status of the Malaysian environment depends on the average number of days with rainfall in a month, and ranges from 70% to 90%^31^ throughout the year. Looking at the number of reported cases over a period of 6 years, the airborne concentration of bacteria, the pH of the soil and enrichment of the soil with nutrients, especially from the areas from where these cases presented (Figure 8), should be investigated further in future experimental studies.

### Epidemiological data

With the exception of one cat with a dubious status of a strictly indoor lifestyle, all other infected cats lived either outdoors or as free roamers (had access to the outside environment) in multi-cat households. A free-roaming lifestyle confirmed the correlation between the occurrence of rhodococcosis and the outdoor lifestyle of cats, as reported in canine and equine cases.^32,33^ About 50–95% of horse farm soil samples are reported to contain *R equi*,^6^ but in these cats there was no history of exposure to such a risk factor, and this was also confirmed by telephone interview. Recently, a study from Denmark reported *R equi* to be one of the resident aerobic microbiota of a healthy nasal cavity in humans, which can later become an opportunistic pathogen in immunocompromised patients.^34^ This could be an interesting topic for future prospective or experimental studies in Malaysia, because of the higher incidence of disease.

The cumulative incidence of *R equi* pneumonia in foals is not dependent on the soil concentration of the virulent strain, but air-borne concentrations have been positively correlated with the disease.^35^ This remained unproven in the current and previously reported feline studies.^4,24,25^ Inhalation of the aerosolised virulent pathogen is one of the main modes of transmission in foals.^15^ One of the most recent case studies where a cat was brought to a horse farm suggested the possibility of the same mode of transmission as the cat was without any history of an injury or wound.^4^ Contrary to all previous reports, the possibility of close contact between cats and horses was less likely for the reported cases in this study, because all the cats lived in urban areas (Figure 8) and contact with farm animals was highly unlikely. In a previous study of cats and dogs on the virulence of documented *R equi* isolates, infected patients, which usually acquire the infection from environmental exposure, were not considered to be a source of human infection. However, it is probable that the cats’ and dogs’ discharges may possess some theoretical risk to immunocompromised owners.

In general, young animals such as foals with a naive immune system, and humans with an inefficient or supressed immune system, are at greater risk of exposure.^36–38^ Efforts have been made previously to correlate the factor of a suppressed immune system in cats with susceptibility to *R equi* infection, but the results were inconclusive owing to the scarcity of cases and limited available data.^4,33^ It was believed that the cutaneous form was the most common manifestation in cats.^4,33^ However, the results of the present study revealed the pulmonary form (36/40 cases) as one of the predominant pictures of disease in cats.

The majority (n = 25/40) of cats in this study were kittens younger than 10 months of age. This observation is similar to what has been reported in equine rhodococcosis, where it is has been described as the most common cause of pneumonia in foals up to 6 months of age.^39^ In the present study, males (n = 27/40) were over-represented and, interestingly, 11/13 females presented with the clinical form of the disease until 10 months of age. A small proportion of cats in this study were adult cats, which seems to be similar to the findings of a comprehensive case study in an adult horse that presented with *R equi*-related pleuropneumonia, where it was concluded that this infection is possibly being overlooked in immunocompetent adult horses.^40^

### Clinical findings

In the present study, of 40 cats, 36 were reported with the pulmonary form of infection, supported by the cytological results of 28 samples with pyogranulomatous inflammation. These results correlated well with the type and presence of characteristic lesions (pyogranulomatous) in equine cases.^16,24^

It was hypothesised that as the intestine is one of the principal sites of predilection in foals, the same would be true for cats; however, the findings of the present study revealed that only 5% of cats had gastrointestinal signs vs 33% in foals.^18^ This suggests that the pathogenesis in cats may be different from that in horses. Previous feline rhodococcosis studies did not report any significant gastrointestinal signs.^24,25^ Passamonti et al^4^ reported diarrhoea as an initial sign, followed by severe respiratory signs, which lead to acute death. There have been reports in horses with subclinical presentation of the pulmonary form with spontaneous recovery, but this mode of pathogenesis and presentation have not been reported in cats to date.^16^

Pyrexia was noted in 34.3% of the cats, but this variable has been considered less important in the modern clinical picture of this infection in horses, after the advancement in disease diagnosis at the subclinical stage.^1^ Lethargy was reported in 20% of cats vs 53% of foals.^41^ Pneumonia with respiratory distress and tachypnoea were predominant clinical presentations in foals,^42^ as well as cats, as shown in the present study.

Regarding haematology, the most common findings were leukocytosis, neutrophilia, band neutrophilia, monocytosis and thrombocytopenia. This pattern correlated well with the reported case studies of foals.^42,43^ Acute septicaemic presentations were the predominant cause of band neutrophilia and monocytosis, often accompanied by left shift.^44^ Chronic stress and increased glucocorticoid activity are indicated by neutrophilia and monocytosis, in combination with lymphopenia and eosinopenia.^44^ Of the cats examined, 19.4% showed lymphopenia, along with neutrophilia and monocytosis. Based on these results and the clinical manifestations in the cats studied, it seems that septicaemia in cats with *R equi* infection did not occur within a short period of time. However, this remains a theory until proper investigations are carried out on the pathogenesis and immune response in cats.

From the biochemistry results, changes in protein levels such as low–normal albumin (n = 36) being a negative phase protein with a mean ± SE value of 28.34 ± 0.849 (range 25–40) and high globulin (n = 36) being a positive acute phase protein with a mean ± SE value of 50.14 ± 1.463 (range 25–45) were prominent findings noted in cats in the present study. Protein levels in equine cases can be influenced by physiological factors, such as age, pregnancy, and clinical infections and inflammatory processes.^40,45^ In cats, these changes have also been attributed to systemic diseases, haematological disorders, chronic infections, inflammatory process and neoplastic disorders.^46^ Hence, these physiological and clinical scenarios highlight the value of protein level changes in cats and horses, especially in ongoing infections and inflammatory processes. An alternative and major differential diagnosis for a low A:G ratio (median value for 36 samples in the present study was 0.6; range: 0.5–1.4) is FIP,^47^ which should be excluded first in this type of clinical finding. Changes in protein levels accompanied by clinical presentations should not prevent clinicians from making a differential diagnosis of *R equi* infection in the prior screening list. The sample size for parameters such as phosphorus, gamma glutamyl transferase, aspartate transaminase, creatine kinase and total bilirubin are too limited to correlate with other species.

### Radiographic examination

To the authors’ knowledge, radiographic abnormalities have not been reported in *R equi* in cats to date. Significant differences have been reported between the *R equi*-positive and *R equi*-negative groups of foals, in terms of pathologies present in thoracic radiographs. Cavitary lesions, nodular lesions, masses and abscesses were worthy of attention in 71.4% of the *R equi-positive* radiographs vs 14.8% of the radiographs of the *R equi*-negative group.^39^ Pleural effusion, a discrete large lung mass and a diffuse interstitial lung pattern in dogs infected by *R equi* have been reported previously.^33^ A cavitary lesion pattern of lung lobes in immunocompromised human cases of pulmonary rhodococcosis have also been reported.^6^ Pulmonary abscess, lymphadenopathy, pleural effusion and pneumothorax were hallmarks of the disease in cats reported in this study. The lack of these signs in feline cases could mask *R equi* infection with other possible infections invading the pulmonary cavity; hence, cytology and isolation of bacteria should be performed for a definitive diagnosis.

The *R equi*-related thoracic radiographic pathologies were quite similar between the group of cats in the present study and the previously reported equine cases.^43^ All cats in this study and in previously reported studies of cats with the cutaneous manifestation of the disease did not show any radiographic changes in lung pattern,^48^ which emphasised the need to understand how the respiratory system can be a major predilection site in feline cases and systemic disease transmission to lung tissue. Post-mortem changes in multiple organs have been reported for this infection in cats,^4^ and the same was noted for cats in the present study.

### Cytological findings

Cytological reports showed a similar type of cellularity and characteristics to previously reported cases in cats and dogs infected with *R equi*.^24,33,48^ Bacterial culture and cytological analysis of the tracheobronchial aspirate in horses has been considered the gold standard.^43^ However, bacterial isolation and the identification process can take up to 72 h before results are obtained and this remains a major challenge in reaching a definitive diagnosis. In cats, cases suspected of having, or with evidence of, pleural effusion should be sampled by thoracocentesis, and chronic infectious pneumonia without thoracic fluid signs should be sampled via bronchoalveolar lavage.^49^ Macrophages were seen in 71% of samples submitted for cytology where phagocytosed bacteria usually presented with activated or foamy/vacuolated cytoplasms,^50,51^ as shown in Figure 4. The histological characteristics of lung tissue were also similar to dogs, including pyogranulomatous inflammation and the presence of intrahistiocytic coccobacillus bacteria.^33^

### Therapy and outcomes

After diagnosis, there are several options for pyothorax case management, but the optimum method is yet to be determined, as management depends on the clinical scenario of acute presentation and the decision-making process of the client and practitioner.^52^ Conventionally, it has been divided into surgical and medical management. Currently, thoracic drainage along with the usage of antimicrobial agents is considered the mainstay of therapy.^53,54^ Thoracic drainage can be considered with single- or repeated-needle thoracocentesis. Other possible options for chest drainage are chest tube thoracostomy, diagnostic and/or therapeutic thoracoscopy and thoracotomy, which can be integrated with a single or multiple lavage, intermittent or continuous suction and intrapleural fibrinolytics administration, and also can be helpful in sample collection.^54^ In the present study, eight cats with the pulmonary form of the disease were managed by chest tube placement: four recovered, two were euthanased and two died during long-term management of the disease.

Because of the acute and dyspnoic presentation most of the cats were placed in a temperature- and humidity-controlled concentrated oxygen chamber for pre-oxygenation, although some cats received flow-by oxygen while being prepared for intravenous (IV) catheter set-up before any further diagnostic and/or therapeutic procedure. Diagnostic and/or therapeutic thoracocentesis was performed in 33 cats presenting with acute respiratory distress while receiving oxygen by face mask or the flow-by method. Concurrent fluid therapy with shock rate was also considered in patients presenting with septic or distributive shock. After this acute management phase, IV antibiotics were administered during the initial course of the disease and were finally replaced by oral antibiotics. All cats that were boarded and required needle thoracentesis more than twice after initial chest tapping were advised to be managed further with chest tube placement. Further indications for chest tube placement included the inabillity to achieve negative pressure at the end of thoracocentesis, if the patient was deteriorating despite multiple needle thoracentesis, and if daily lavage and continuous or intermittent suction was required, and after thoracotomy surgery.^55^

In antimicrobial susceptibility testing in vitro there might be a wide range of antibiotics that can be effective, but the use of lipophilic antibiotics (ie, macrolides), especially erythromycin and rifampin in combination, has been recommended in foals because of the potential in vivo synergism, penetration in caseous material and macrophages, and remarkable improvement in the clinical status of the infected patients.^56^ The same seems to be an effective combination for cats, given the outcome after their use in pre- and post-diagnostic management. In the past, potentiated amoxicillin and tetracyclines were relatively better choices for feline rhodococcosis (used against any serotype).^24^ In the present study, they showed very poor susceptibility. Macrolides, gentamicin and enrofloxacin showed similar results,^24^ noted in this study. The major difference observed in treatment outcome was that 3/4 cats (cutaneous form) in this study died without any appropriate response to antibiotics, compared with the cutaneous cases in a previous study,^24^ where all cats recovered (n = 5). Contamination could be a suggested source of infection for the cutaneous form, without any concrete evidence of the immune status of the cats in the present study.

The average duration of therapy in responding cats was 6 weeks in this study. Unlike natural infection in foals, the immunosuppression component has been observed in human cases and in a mouse model.^48^ The duration of therapy was variable in different hosts from various studies, depending on the virulence of the strain, and the response and resistance status of the antibiotics used.^2,19,56,57^

Despite the use of multiple antibiotics to combat infection, the survival rate was rather unsatisfactory. Client compliance, quality of treatment and initial management could have a significant impact on survival rates, but limited susceptibility tests and unknown virulence typing of *R equi* hinders clinicians in making better choices in their selection of antibiotics, and the most effective combination.

## Conclusions

Pulmonary rhodococcosis is an extremely rare condition, but it seems to be an emerging disease in the Malaysian cat population. A complete history and thorough physical examination are mandatory to investigate the exposure to infection. Thoracic radiographs are effective in describing the clinical signs and deciding on disease management. Bacterial cultures along with cytology are the key tests in making a definitive diagnosis in cats. The effectiveness of the therapy depends on the susceptibility of the antibiotic combination being used for treatment. The overall prognosis appears to be poor, based on the results of this study. Much is still unknown regarding immune status-related predisposing factors, epidemiology, virulence and pathogenesis of *R equi* in cats. Further clinical, prospective and experimental studies are necessary to elucidate the various aspects of *R equi* infection in cats.
